# A protocol for identifying universal reference genes within a genus based on RNA-Seq data: a case study of poplar stem gene expression

**DOI:** 10.48130/forres-0024-0017

**Published:** 2024-06-01

**Authors:** Qi Xie, Umair Ahmed, Cheng Qi, Kebing Du, Jie Luo, Pengcheng Wang, Bo Zheng, Xueping Shi

**Affiliations:** 1 National Key Laboratory for Germplasm Innovation & Utilization of Horticultural Crops, Huazhong Agricultural University, Wuhan 430070, China; 2 College of Horticulture and Forestry Sciences, Huazhong Agricultural University, Wuhan 430070, China; 3 Poplar Research Center, Huazhong Agricultural University, Wuhan 430070, China; 4 Hubei Engineering Technology Research Center for Forestry Information, Huazhong Agricultural University, Wuhan 430070, China

**Keywords:** Reference genes, *Populus*, Gene expression, Transcriptome, RT-qPCR, Abiotic stress, Stem development

## Abstract

Real-time quantitative reverse transcription polymerase chain reaction (RT-qPCR) plays a crucial role in relative gene expression analysis, and accurate normalization relies on suitable reference genes (RGs). In this study, a pipeline for identifying candidate RGs from publicly available stem-related RNA-Seq data of different *Populus* species under various developmental and abiotic stress conditions is presented. DESeq2's median of ratios yielded the smallest coefficient of variance (CV) values in a total of 292 RNA-Seq samples and was therefore chosen as the method for sample normalization. A total of 541 stably expressed genes were retrieved based on the CV values with a cutoff of 0.3. Universal gene-specific primer pairs were designed based on the consensus sequences of the orthologous genes of each *Populus* RG candidate. The expression levels of 12 candidate RGs and six reported RGs in stems under different abiotic stress conditions or in different *Populus* species were assessed by RT-qPCR. The expression stability of selected genes was further evaluated using ΔCt, geNorm, NormFinder, and BestKeeper. All candidate RGs were stably expressed in different experiments and conditions in *Populus*. A test dataset containing 117 RNA-Seq samples was then used to confirm the expression stability, six candidate RGs and three reported RGs met the requirement of CV ≤ 0.3. In summary, this study was to propose a systematic and optimized protocol for the identification of constitutively and stably expressed genes based on RNA-Seq data, and Potri.001G349400 (*CNOT2*) was identified as the best candidate RG suitable for gene expression studies in poplar stems.

## Introduction

RNA sequencing (RNA-Seq) technology has emerged as a powerful tool in transcriptomic studies, offering high accuracy, sensitivity, and resolution^[1]^. Unlike traditional methods, RNA-Seq does not rely on prior knowledge of specific RNA molecules, making it effective for identifying unknown RNAs^[2]^. In RNA-Seq, total RNA or specific RNA fragments are isolated from samples representing different biological conditions or replicated under similar conditions. Recent advancements in next-generation sequencing (NGS) technology have made RNA-Seq the preferred approach for gene expression studies, thanks to its cost-effectiveness and technological improvements. This sequence-based method has revolutionized transcriptome research, enabling various applications, including the analysis of strand-specific expression, the detection of transcript fusions and alternative splicing isoforms, and the characterization of unknown cell types (through single-cell RNA sequencing)^[3,4]^.

RNA-Seq also enables better discovery of differentially expressed genes (DEGs) in various biological tissues and growth conditions, and may be able to provide high genome coverage even for genes with low expression levels^[5]^. RNA-Seq analysis measures transcript abundance by quantifying the fragments generated and the number of reads corresponding to each transcript. Since the total RNA content in a sample is unknown, data normalization is essential. Normalization methods include individual normalization based on the total number of reads and transcript lengths in each sample, resulting in Reads Per Kilobase of exon per Million mapped reads (RPKM) or Fragments Per Kilobase of transcript per Million mapped reads (FPKM) values^[6]^. Alternatively, normalization can be achieved using different methods such as the Trimmed-Mean of M-values (TMM), DESeq2's median of ratios, Transcript Per Million (TPM) and Upper Quartile (UQ). Similar to RPKM, TPM doesn't utilize read information from all samples for normalization^[7,8]^.

In terms of gene expression analysis, RNA-Seq experiments primarily focus on identifying DEGs in specific biological conditions. However, apart from DEGs, there are numerous genes known as constitutively expressed genes (CEGs) that exhibit consistent expression across different cells or developmental stages, regardless of environmental conditions. For instance, a study on rice revealed that 22.7% of transcripts were expressed by CEGs in 39 different rice tissues^[9]^. Surprisingly, recent studies suggest that CEGs exhibit variable expression under different conditions and are used as reference genes (RGs)^[10,11]^. An ideal RG is one that remains unaffected by any experimental condition, shows stable expression, has no pseudogenes, and has a mid-range of quantification cycles or Cq values (Cq = 15–25) in real-time quantitative reverse transcription polymerase chain reaction (RT-qPCR)^[12]^. So technically speaking, every RG is CEG but not every CEG is RG^[13]^.

RT-qPCR is a reliable technique for transcript detection and measurement. To ensure accurate RT-qPCR results, it is crucial to select suitable RGs for normalization, following the guidelines outlined in the MIQE (Minimum Information for Publication of Quantitative Real-Time PCR Experiments) standards^[14]^. RGs should ideally display constant expression levels across various plant tissues, developmental stages, or physiological conditions. They should remain unaffected by external treatments and can be used without the need for stability validation^[15]^. However, studies focusing on RG validation and comprehensive exploration of transcriptome data in model plants have shown that the accuracy of endogenous controls can be significantly influenced by factors such as plant species, the specific cells/tissues/organs under investigation, and growth conditions^[16]^. Therefore, the selection of appropriate RGs is a critical step in normalizing RT-qPCR data, as an incorrect selection may lead to ambiguous or even incorrect results^[17,18]^.

Traditionally, RGs in many horticultural plants were selected from cellular housekeeping genes in the absence of large genomic datasets^[12]^. Examples include *elongation factor-1α* (*EF-1A*) in zucchini, *actin* in poplar, and *ubiquitin conjugating-enzyme* (*UBC*) in banana^[12]^. However, it has been observed that certain RGs exhibit significant expression variability among different conditions and tissue types^[19]^. Moreover, even within species, different RG candidates may show varying expression stability under specific experimental conditions or tissue types^[20,21]^. Therefore, it is crucial to validate the expression stability of candidate RGs before utilizing them for data normalization. Only those RG candidates that have undergone rigorous validation for expression stability can be considered reliable RGs for specific conditions or tissue types^[22]^. Several statistical tools, such as BestKeeper, geNorm, and NormFinder, are commonly employed to identify the most suitable candidate RGs under specific experimental settings^[12]^. These tools aid in selecting RGs that exhibit minimal expression variability across various conditions or tissues.

Forest trees and horticultural plants encompass a diverse range of species, many of which are still in the early stages of genomic and functional genomic research. The emergence of RNA-Seq technology has significantly advanced gene annotation, expression profiling, and functional studies in these plants. The availability of extensive RNA-Seq data sets provides valuable resources for selecting suitable RGs across different species and under various experimental conditions^[23−25]^. Poplar, a widely distributed tree species with significant applications in wood production, environmental protection, and urban greening, serves as an important model plant for woody species^[26]^. Extensive research has been conducted on poplar stem growth and development, leading to the generation of large-scale transcriptome data sets^[27−29]^. In this study, the objective is to retrieve and assess the quality of publicly available transcriptome data sets related to poplar stem tissues. Subsequently, we aim to predict and evaluate the best candidate RGs for gene expression analysis in stems of various *Populus* species. The expression stability of these candidate RGs under different stress conditions will be validated using RT-qPCR, leading to the identification of reliable RGs specific to poplar stems. Furthermore, based on this case study, we intend to establish a comprehensive pipeline for the development of RGs for accurate gene expression normalization using RNA-Seq data.

## Materials and methods

### Workflow for identifying RGs

**Figure 1:**
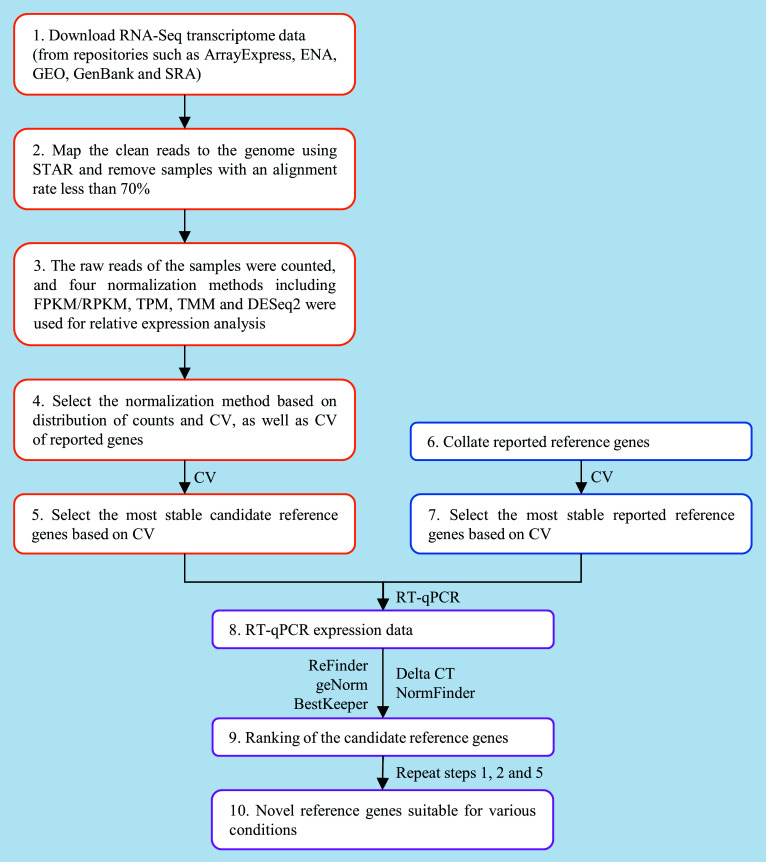
Workflow for selecting novel reference genes (RGs) based on RNA-Seq data.

The workflow for identifying universal RGs within a genus based on RNA-Seq data is shown in Fig. 1, using RNA-Seq data downloaded from public databases for poplar stem gene expression.

### Download RNA-Seq transcriptome data for poplar stem samples

To obtain the RNA-Seq data of poplar stems, a comprehensive literature search was conducted to identify relevant studies that reported RNA-Seq experiments on poplar stem samples. A dataset comprising 298 RNA-seq samples focusing on poplar stem research was compiled as the training dataset for this study (Supplemental Dataset 1). These samples were derived from 10 distinct experimental groups, each representing different biological aspects of poplar stem. The groups included experiments examining different internodes of poplar^[27]^, various stem tissues such as phloem, xylem, fiber cells, and ray cells^[30,31]^, the entire developmental stage spanning from phloem to xylem^[32]^, abiotic stress treatments such as low temperature, heat, drought, and salt^[33]^, gravity stimulation by placing plants horizontally^[34]^, different growth areas^[35]^, biological stress treatments^[36]^, overexpression of miR397a^[37]^, and transgenic lines treated with ACC (1-aminocyclopropane-1-carboxylic acid)^[38]^ (Supplemental Table S1). Accession numbers associated with the identified studies were then obtained. Subsequently, the RNA-Seq data corresponding to these accession numbers were downloaded from the National Center for Biotechnology Information (NCBI: www.ncbi.nlm.nih.gov) or EMBL's European Bioinformatics Institute (EMBL-EBI: www.ebi.ac.uk) databases. Only RNA-Seq data from poplar stem samples with a minimum of two biological replicates were included in the analysis (Supplemental Table S1).

### Quality control, filtering, and mapping of the RNA-Seq data

The RNA-Seq data used in this study were obtained from sub-databases of NCBI, including GenBank, GEO, and SRA, as well as from EMBL-EBI, which includes ArrayExpress and ENA (European Nucleotide Archive). For the SRA data, the sratoolkit software v2.9.2 (https://ftp-trace.ncbi.nlm.nih.gov/sra/sdk/) was employed to convert the SRA data into FASTQ format before subsequent analysis. The default parameters were used for single-end (SE) sequencing data during the conversion process, while additional parameters (--split-3) were added for paired-end (PE) sequencing data to appropriately handle the data. The data downloaded from the EMBL-EBI database were already in FASTQ format and could be directly used for subsequent analysis.

The raw data quality was assessed using FastQC v0.10.1 (www.bioinformatics.babraham.ac.uk/projects/fastqc). Subsequently, Trimmomatic v0.32^[39]^ was applied for the removal of adaptor contaminants and low-quality reads. The parameter settings for SE and PE sequencing data were as follows: TruSeq3-SE-2.fa:2:30:10 LEADING:3 SLIDINGWINDOW:5:20 MINLEN:50 and TruSeq3-PE-2.fa:2:30:10 LEADING:3 SLIDINGWINDOW:5:20 MINLEN:50, respectively^[40]^. These steps ensured that the RNA-Seq data used in the subsequent analysis was of high quality and free from artifacts and noise.

Following the trimming and filtering of the raw data to obtain clean reads, the clean reads were mapped to the *P. trichocarpa* v3.0 genome using STAR v2.3.0e_r291^[41]^. The mapping process utilized the '--readFilesCommand zcat --quantMode TranscriptomeSAM' settings. By mapping the clean reads to the reference genome, the total number of reads in each sample and the number of reads that successfully mapped to the genome were determined. The alignment rate, representing the proportion of mapped reads to the total number of reads, was calculated for each sample. Samples with an alignment rate exceeding 70% were retained for subsequent analysis.

Upon mapping the clean reads to the genome, a BAM file was generated for each sample. The BAM files contained information about the alignment positions of the reads, enabling the determination of the relative expression levels of all genes in each sample. This step provided the necessary data for further analysis and characterization of gene expression patterns in the poplar stem samples.

### Comparison of RNA-Seq normalization methods and assessment of gene expression

Four normalization methods, including FPKM/RPKM, TPM, TMM, and DESeq2's median of ratios, were utilized to assess and compare the impact of different normalization approaches on the gene expression analysis and to identify the most appropriate method for the RNA-Seq datasets. The BAM files generated from the previous mapping step were analyzed using the featureCounts program, a part of the Subread package^[42]^, to obtain the unnormalized raw count (RC) of each gene in each sample. For TMM normalization of the RCs, the edgeR package^[43]^ was utilized, while DESeq2 package^[44]^ was employed for DESeq2's normalization. To perform FPKM/RPKM and TPM normalization, the RSEM (RNA-Seq by Expectation Maximization) software^[45]^ was used, applying default parameters. These normalization methods were applied to the RCs, resulting in normalized expression values that can be compared across samples.

To compare the effects of FPKM/RPKM, TPM, TMM, and DESeq2's median of ratios on the normalization of RC, a bar graph was generated to depict the expression levels of all genes from the lower quartile to the upper quartile values for each method. The relative expression level of each gene was calculated as log_2_(value+1) to mitigate any outliers resulting from zero values. The Coefficient of Variance (CV) was then calculated for the relative expression level of each gene across all samples. To investigate the variability and consistency of gene expression across RC, FPKM/RPKM, TPM, TMM, and DESeq2's median of ratios methods, a box plot was utilized to visualize the CV distribution of the relative expression levels for all genes.

Moreover, the CV of the relative expression levels of the reported RGs were used to assess the accuracy and reliability of RC, FPKM/RPKM, TPM, TMM, and DESeq2's median of ratios methods. The CV provides a measure of the variation in gene expression across different samples and allows us to assess the stability and consistency of the RGs under each normalization method.

The reported RGs in poplar stems had been extensively collected through a literature review^[24,46−48]^. After removing duplicate genes, the relative expression levels of 30 reported RGs were evaluated in all samples (Supplemental Table S2). The CV values and the average CV values of their relative expression levels before and after normalization were then calculated using the four different normalization methods. Boxplots were generated to illustrate the distribution of CV values and the means of CV values.

After normalizing the data using four normalization methods, the distribution of relative expression levels as well as the distribution of CV values for all *Populus* genes (42,950 genes) were comprehensively analyzed. The CV distribution and the mean values for the relative expression levels of the 30 reported RGs were also examined. The optimal normalization method was selected based on the above analyses. The relative expression data of all samples were then processed using the selected normalization method for further analysis.

### Selection and evaluation of candidate RGs based on RNA-Seq

Genes with a CV value of ≤ 0.3 in their relative expression levels across all samples, as determined using the optimal normalization method, were considered to have stable expression. These genes were classified as stably expressed genes in this study. The distribution densities of the relative expression levels for all genes, the stably expressed genes, and the 30 reported RGs were statistically analyzed. Available Gene Ontology (GO) and Kyoto Encyclopedia of Genes and Genomes (KEGG) annotation information for all genes in the *P. trichocarpa* v3.0 genome were used. To further investigate the functional enrichment of the stably expressed genes and the 30 reported RGs, GO, and KEGG enrichment analysis were performed using TBtools software^[49]^.

Based on the CV values of the relative expression levels of genes across all samples, the top 12 genes with the smallest CV values were selected as candidate RGs for this study. To further confirm the expression stability of the 12 candidate RGs, RT-qPCR experiments were performed and the results were compared with the six reported RGs with the smallest CV in relative expression levels.

### Plant material and growth conditions

Hybrid poplar 717 (*P. tremula* × *P. alba* clone INRA 717-1B4, hereafter referred to as poplar 717) plants were cultured in 250 mL glass bottles containing 35 mL of 1/2 Murashige and Skoog (MS) medium (Phytotech, Lenexa, USA) supplemented with 0.75% (w/v) agar and 3% (w/v) sucrose. The bottles were placed in a growth chamber with a photoperiod of 16 h of light and 8 h of darkness at a temperature of 25 °C for a duration of two months. The tissue-cultured plants were then transplanted into pots 8.5 cm in diameter and 14 cm high, filled with a soil mixture of three parts peat, two parts base soil, and one part vermiculite. The plants were grown in a greenhouse at a temperature of 28 ± 5 °C for three months. Five *Populus* cultivars including *P. simonii*, *P. deltoides*, hybrid poplar clone 717 (*P. tremula × P. alba* L.), *P. alba* and *P. adenopoda* were grown in the field of the campus of Huazhong Agricultural University in Wuhan, China.

### Stress treatments

Poplar 717 plants of uniform size (100−110 cm) were selected and subjected to various abiotic stresses (Supplemental Table S3). Salt stress was induced by exposing plants to 150 mM NaCl solution for 2 d (D2), 4 d (D4), and 6 d (D6). The plants were watered with the NaCl solution every 24 h. The drought stress treatment adopted the process of simulating the gradual drying of soil. Three groups of plants were withheld water for 5 d (D5), 10 d (D10), and 15 d (D15) to achieve mild, moderate, and severe drought conditions, respectively. The control group (D0) was watered regularly according to evaporative needs. Relative soil moisture was measured using a FieldScout^TM^ TDR 300 Soil Moisture Meter (Spectrum Technologies, USA). For shade stress, plants were covered by black nylon mesh to achieve 70%−80% shading. The time points for shade stress were D0, D5, D10 and D15. The light intensity at each time point was measured using a TM-205 Auto Ranging Lux/Fc Light Meter (TENMARS, Taiwan). In the above stress treatments, the physiological parameters such as net photosynthetic rate (Pn), stomatal conductance (gs), intercellular CO_2_ (Ci), and transpiration rate (Tr) of the poplar 717 plants were measured using a LI-6400XT portable photosynthesis system (LI-COR, USA). Gravity stress was induced by bending the plants to investigate tension wood and opposite wood at D2 and D14 time points. For each time point of each treatment, the number of sample replicates was three.

### Design of primers for RT-qPCR experiment

Primers for RT-qPCR experiments were designed to ensure gene specificity within species and generality among species, and the following primer design steps were performed. The primary transcript sequences of the RGs from *P. trichocarpa* were used to perform blastn searches (-evalue: 1e-5, -num_alignments: 5) to obtain their homologous transcripts in *P. trichocarpa*, *P. euphratica*, *P. deltoides*, *P. tremula* and *P. alba*. To select the most homologous genes for each RG, a phylogenetic analysis of RG and its homologs was performed using MEGA X^[50]^ with a maximum likelihood method and a bootstrap value of 1,000. To obtain the consensus nucleotide sequence of each RG, multiple sequence alignment was then performed between homologous transcript sequences from different *Populus* species. Based on the consensus sequence, universal primer pairs for each RG were designed using Primer3 software (https://primer3.ut.ee/) with the following criteria: primer length of 18−27 nt, primer melting temperature (T_m_) of 59−63 °C, and product size of 100−300 bp. Finally, mfeprimer-3.2.0 software^[51]^ was used to perform gene-specific detection of the designed primers in the aforementioned five genomes.

### Total RNA extraction and RT-qPCR

After the stress treatments, samples were taken from the 9^th^ internode (IN9) at the branch top of each plant. Of the field-grown poplar trees, the young (IN3) and mature stems (IN9) were collected from each cultivar. The samples were immediately placed in liquid nitrogen and stored at −80 °C for RT-qPCR analysis. All plants were sampled with three biological replicates.

The total RNA was extracted from the young and mature stem tissues of poplar plants using the 2×CTAB method as previously described^[52]^. The quality and quantity of the extracted RNA was assessed using a NanoDrop^TM^ 2000 spectrophotometer (Thermo Scientific, USA). To generate complementary DNA (cDNA), the PrimeScript^TM^ RT Reagent Kit with gDNA Eraser (TaKaRa, Dalian, China) was used following the manufacturer's instructions. Reactions of cDNA synthesis were performed in 20 µL volumes. The resulting cDNA was diluted 20-fold with ultrapure water and served as a template for RT-qPCR analysis.

For RT-qPCR, ChamQ^TM^ SYBR® qPCR Master Mix (High ROX Premixed) from Vazyme (Nanjing, China) was used following the manufacturer's instructions. The RT-qPCR was conducted on a Light Cycler® 480 instrument II from Roche (USA) using white 384-well plates. The gene expression levels of the candidate RGs were evaluated.

To determine the amplification efficiency (E) and R^2^ value for all primer pairs, a 6-point standard curve was generated by performing 20× dilutions of cDNA. The amplification efficiency (E) was calculated as E = 10^(−1/slope of the standard curve), and the R^2^ value was determined. All RT-qPCR experiments were performed with three biological replicates and two technical replicates.

### Stability evaluation of candidate RGs

The expression stability of the candidate RGs and reported RGs were evaluated using Ref-Finder (www.leonxie.com/referencegene.php), which incorporates the delta-Ct, BestKeeper, Normfinder, and geNorm algorithms. The evaluation was based on the average Ct values obtained from RT-qPCR experiments. By considering the comprehensive geometric stability ranking, candidate RGs with the most stable expression were identified and selected as novel RGs for poplar stems.

To evaluate the expression stability of the candidate RGs and reported RGs, a test dataset of 117 RNA-Seq samples was compiled, focusing on poplar stem studies (Supplemental Table S4). The samples came from nine different experimental groups recently uploaded to the public databases and represent various biological aspects of poplar stems, including flexure treatment^[53]^, auxin treatment^[54]^ and transgenic studies on *ERF15*^[55]^, *miR408*^[56]^ and *PtrVCS2*^[57]^. The test dataset was downloaded and processed as previously described for the training dataset. After normalizing the data by using the DESeq2's median of ratios method, distributions, and CV values of relative expression levels were used to assess the expression stability of the 12 candidate RGs and six reported RGs in the test dataset.

### Data analysis and plotting

Data statistics and analysis were performed using the R programming language. Specifically, R language packages, such as ggplot and ggtree, were utilized for data visualization and plotting to create high-quality graphics and visualize data in a clear and informative manner.

## Results

### Collection and screening of poplar transcriptome data and collation of reported RGs

Through a comprehensive review of relevant literature on poplar transcriptome sequencing, a dataset comprising 298 RNA-seq samples focusing on poplar stem research was compiled for this study (Supplemental Dataset 1). These samples provide a comprehensive representation of poplar stem development, enabling the identification and evaluation of candidate RGs. The 298 samples were aligned to the *Populus trichocarpa* v3.0 genome, resulting in 294 samples with an alignment rate exceeding 70% (Supplemental Dataset 2). Among these, samples K5_20 and K5_28 had alignment rates higher than 80% (84.80% and 84.49% respectively), but were excluded from further analysis due to their low read counts^[41]^. Consequently, transcriptome data from a total of 292 samples were retained for subsequent analysis in this study.

In addition, a collection of 37 reported RGs in poplar stems was compiled. After removing duplicate genes based on their gene ID numbers, a set of 30 reported RGs was obtained for further analysis (Supplemental Table S2).

### Selection of normalization method

**Figure 2:**
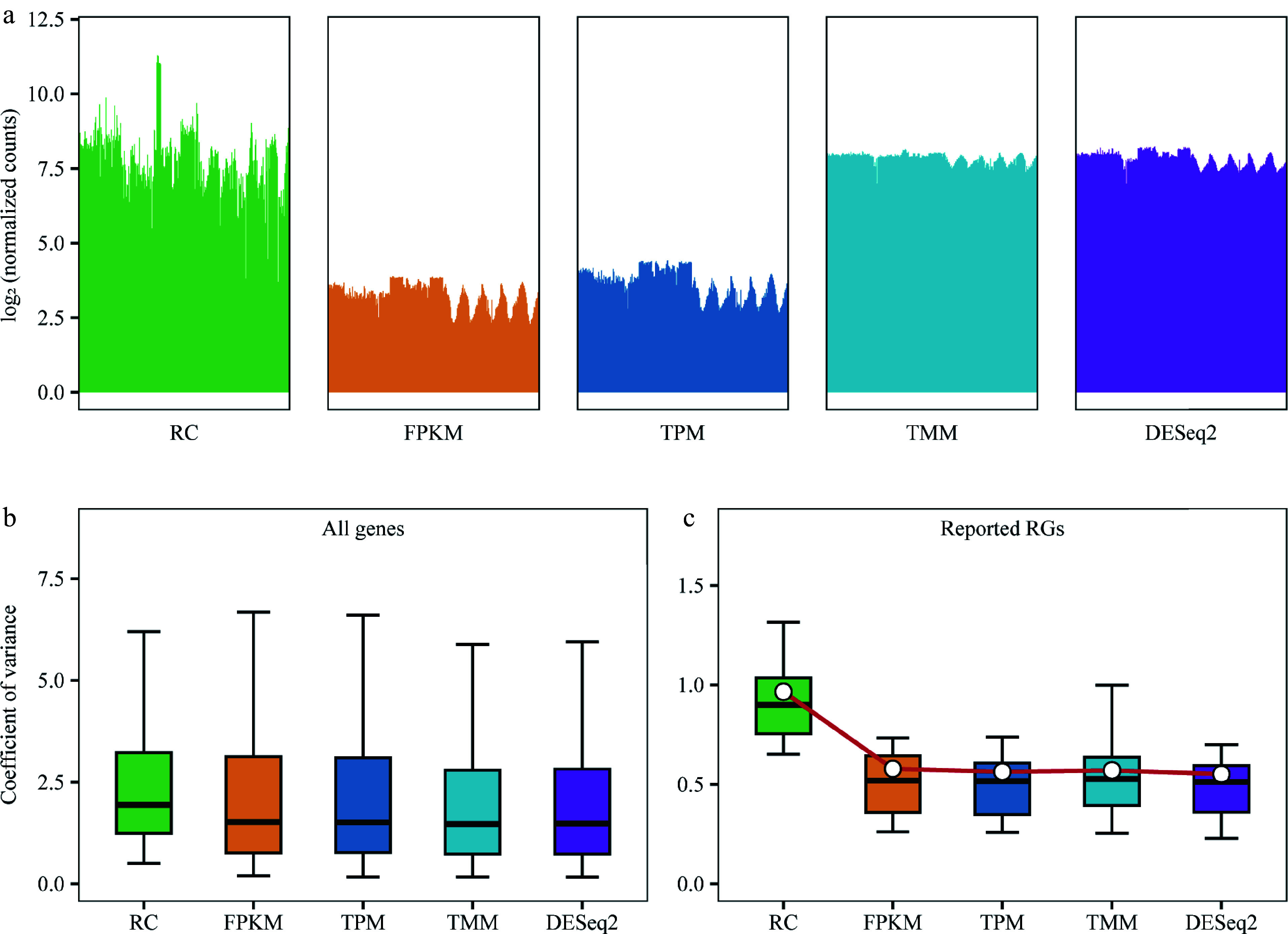
Comparison of normalization methods. RC (raw counts) represents raw counts without normalization, while FPKM, TPM, TMM, and DESeq2 are results after normalization using four different methods. (a) Distribution of counts for all genes in each sample before and after normalization using 292 samples. Bar plots are employed instead of box plots, with the upper limit of the bar representing the upper quartile and the lower limit representing the lower quartile of the data. (b) Box plot demonstrates the coefficient of variance (CV) of gene expression levels for all genes before and after normalization, utilizing data from 292 samples. (c) Box plot depicting the CV for 30 previously reported reference genes (RGs) after different normalizations across all 292 samples, with the central white point indicating the mean CV.

By analyzing the upper and lower quartile values of gene expression levels in the 292 samples before and after normalization, and presenting the results in bar graphs, the differences in gene expression levels among samples were reduced after normalization with RPKM/FPKM, TPM, TMM, and DESeq2's median of ratios compared to without normalization (RC). Specifically, the distribution of gene expression levels between samples became more similar and less variable after DESeq2 and TMM normalization, indicating that these methods performed better than RPKM/FPKM and TPM (Fig. 2a). Furthermore, the CV of the relative expression of all genes in the 292 samples were examined. After normalization with RPKM/FPKM, TPM, TMM, and DESeq2's median of ratios, the median CV values were 1.52, 1.51, 1.47, and 1.48, respectively, with TMM and DESeq2's median of ratios exhibiting more favorable effects compared to RPKM/FPKM and TPM (Fig. 2b). To evaluate the effect of different normalization methods, the changes in the expression levels of the 30 reported RGs were analyzed. The median and mean CVs for unnormalized RC were 0.90 and 0.97, respectively. After normalization with RPKM/FPKM, TPM, TMM, and DESeq2's median of ratios, the median CVs were 0.52, 0.52, 0.53, and 0.51, respectively; and the mean CVs were 0.58, 0.56, 0.57, and 0.55, respectively (Fig. 2c).

DESeq2's median of ratios and TMM showed similar effects in normalizing the expression levels of the 30 reported RGs in poplar. However, DESeq2's median of ratios yielded the smallest CV values and was therefore chosen as the method for sample normalization in the subsequent steps.

### Screening and functional annotation of stably expressed genes

**Figure 3:**
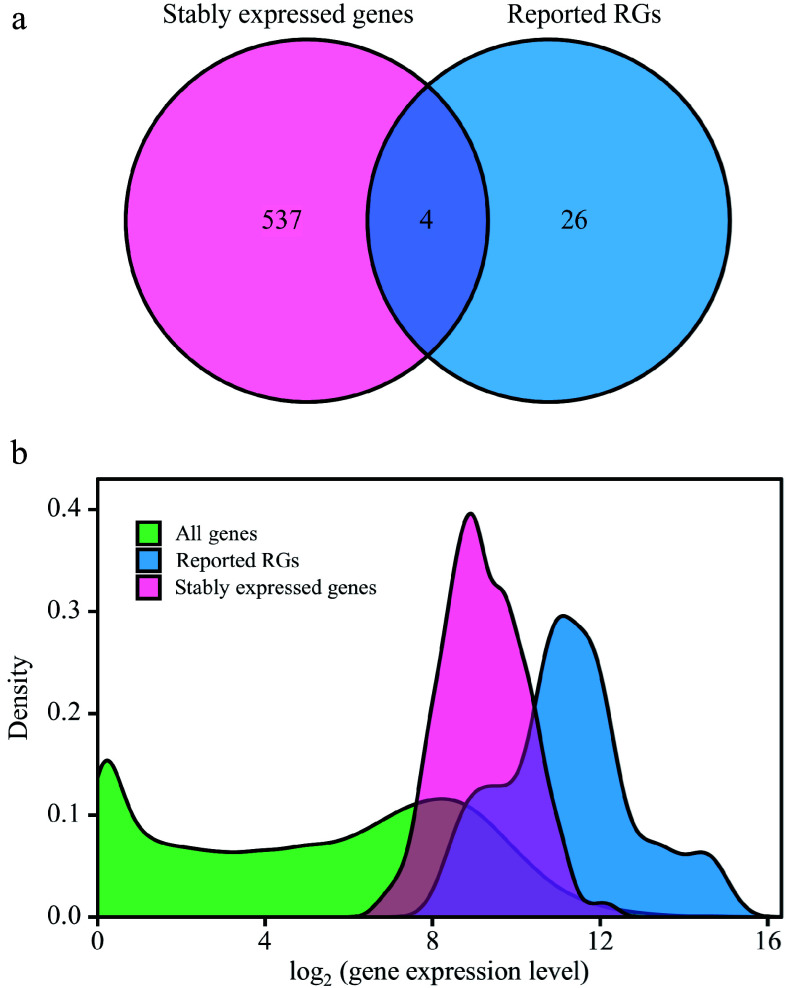
Comparative analysis between stably expressed genes and reported reference genes (RGs). This figure presents a comparison of expression patterns between stably expressed genes and previously reported RGs. (a) Venn diagram illustrates the overlap between stably expressed genes (with CV ≤ 0.3) identified from 292 RNA-Seq samples and the reported RGs. (b) The density distribution of gene expression levels is depicted for all genes in the *Populus* genome, stably expressed genes, and reported RGs. The X-axis represents log2(counts + 1), and the Y-axis represents gene density.

To screen out the RGs suitable for various poplar species and experimental treatments, the RNA-Seq data of the 292 samples were further analyzed. After RC was normalized by the DESeq2's median of ratios method, the mean, standard deviation (SD), and CV of the relative expression levels of each gene were calculated. A total of 541 stably expressed genes (CV ≤ 0.3) were screened, including four reported RGs (*PP2A-2*, *EIF-A*, *CDPK* and *ATPase*) (Fig. 3a, Supplemental Dataset 3). The log values of the relative expression levels' mean of the 541 stably expressed genes ranged from 5.91 to 13.03; and for the 30 reported RGs, the log values ranged from 7.17 to 16.09 (Fig. 3b). This indicates that the stably expressed genes identified in this study have similar expression levels to the reported RGs. GO and KEGG enrichment analyses were performed on the stably expressed genes and the reported RGs. The results indicate that the stably expressed genes identified in this study share functional similarities with the reported RGs (Supplemental Dataset 4).

### Selection of candidate RGs

**Figure 4:**
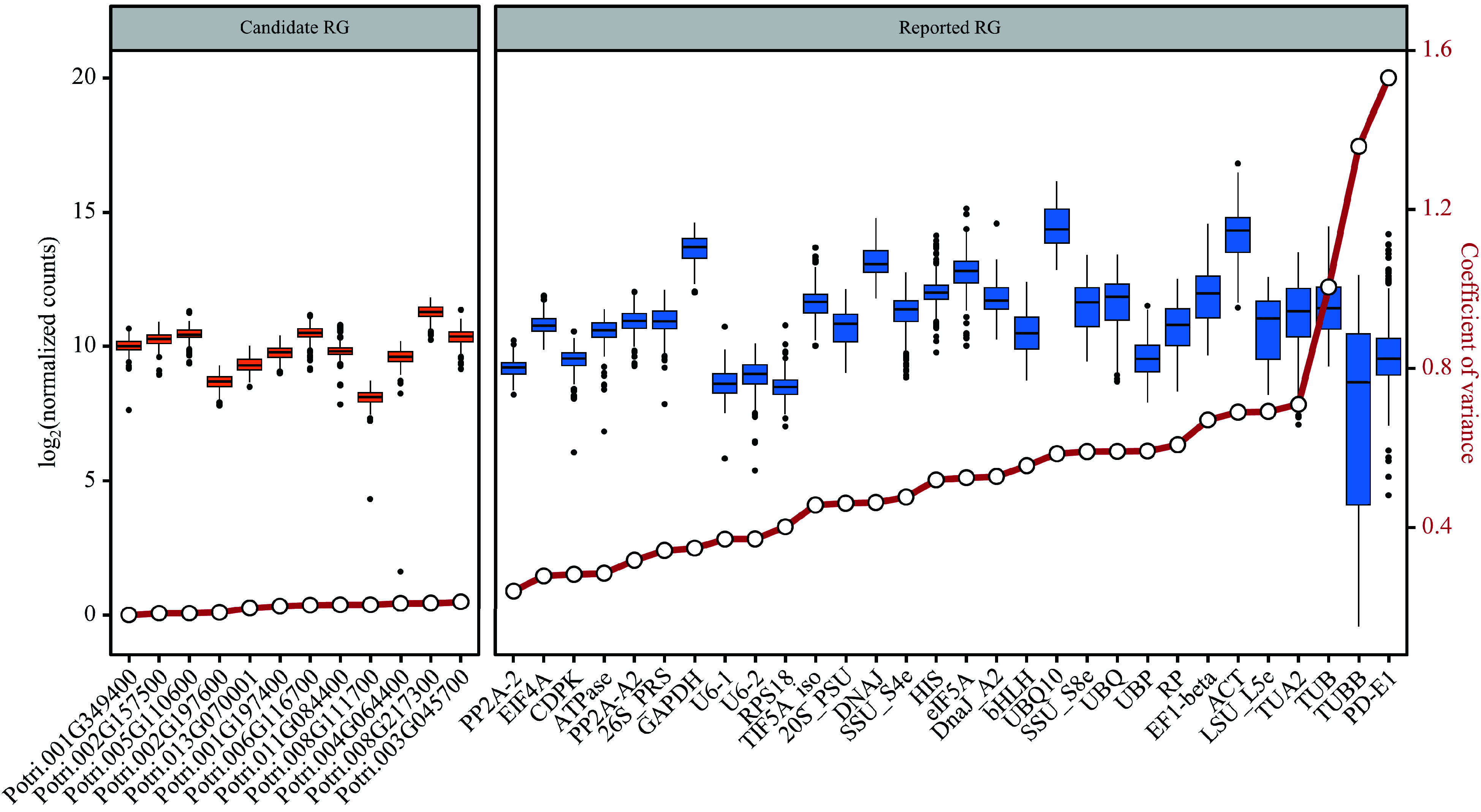
Assessment of candidate and reported reference genes (RGs) stability based on RNA-Seq data. Box plots are employed to display the distribution of expression levels for 12 candidate RGs and 30 reported RGs. The left side shows the expression distribution of candidate RGs, while the right side shows the expression distribution of reported RGs. The small circles on the red lines represent the coefficient of variance (CV) for the gene expression levels.

From the 541 stably expressed genes, 12 genes (Table 1) with the smallest CV values among the 292 samples were selected as candidate RGs, based on the normalization method of DESeq2's median of ratios. Comparing the stability of expression levels between these candidate RGs and the reported RGs, the former had CV values ranging from 0.16 to 0.20, and the latter had CV values ranging from 0.23 to 1.53. The expression of the candidate RGs showed better stabilities (lower CV values) than the reported RGs in the 292 samples (Fig. 4). Furthermore, using the six reported RGs with the smallest CV values as comparisons, the expression stability of 12 candidate RGs was verified by RT-qPCR analysis.

**Table 1:** Information of candidate reference genes (RGs) with the smallest coefficient of variance (CV) values among the 292 RNA-Seq samples.

Gene ID	Gene name	Description	CV
Potri.001G349400	*CNOT2*	CCR4-NOT transcription complex subunit 2	0.168
Potri.002G157500	*RH8*	Similar to DEAD/DEAH box helicase	0.172
Potri.005G110600	*VPS35*	Vacuolar protein sorting-associated protein 35	0.173
Potri.002G197600	*FIP37.1*	Similar to ARABIDOPSIS THALIANA FKBP12 INTERACTING PROTEIN 37	0.175
Potri.013G070001	*NA*	UDP-glucose pyrophosphorylase	0.186
Potri.001G197400	*Pt-UBP6.2*	Similar to UBIQUITIN-SPECIFIC PROTEASE 6	0.190
Potri.006G116700	*U2AF1*	Splicing factor U2AF 35 kDa subunit	0.193
Potri.008G111700	*NA*	Predicted hydrolases of HD superfamily	0.194
Potri.011G084400	*CUL4*	Similar to hypothetical protein	0.194
Potri.004G064400	*NA*	Similar to ankyrin protein kinase	0.198
Potri.008G217300	*Pt-CUL1.4*	Similar to cullin-like protein1	0.199
Potri.003G045700	*Pt-ATRLI1.2*	Similar to RNase L inhibitor protein; putative	0.202
NA: not available. The estimated CVs were based on the normalization method of DESeq2's median of ratios.			

### Design of RT-qPCR primers and validation of amplification efficiency

**Figure 5:**
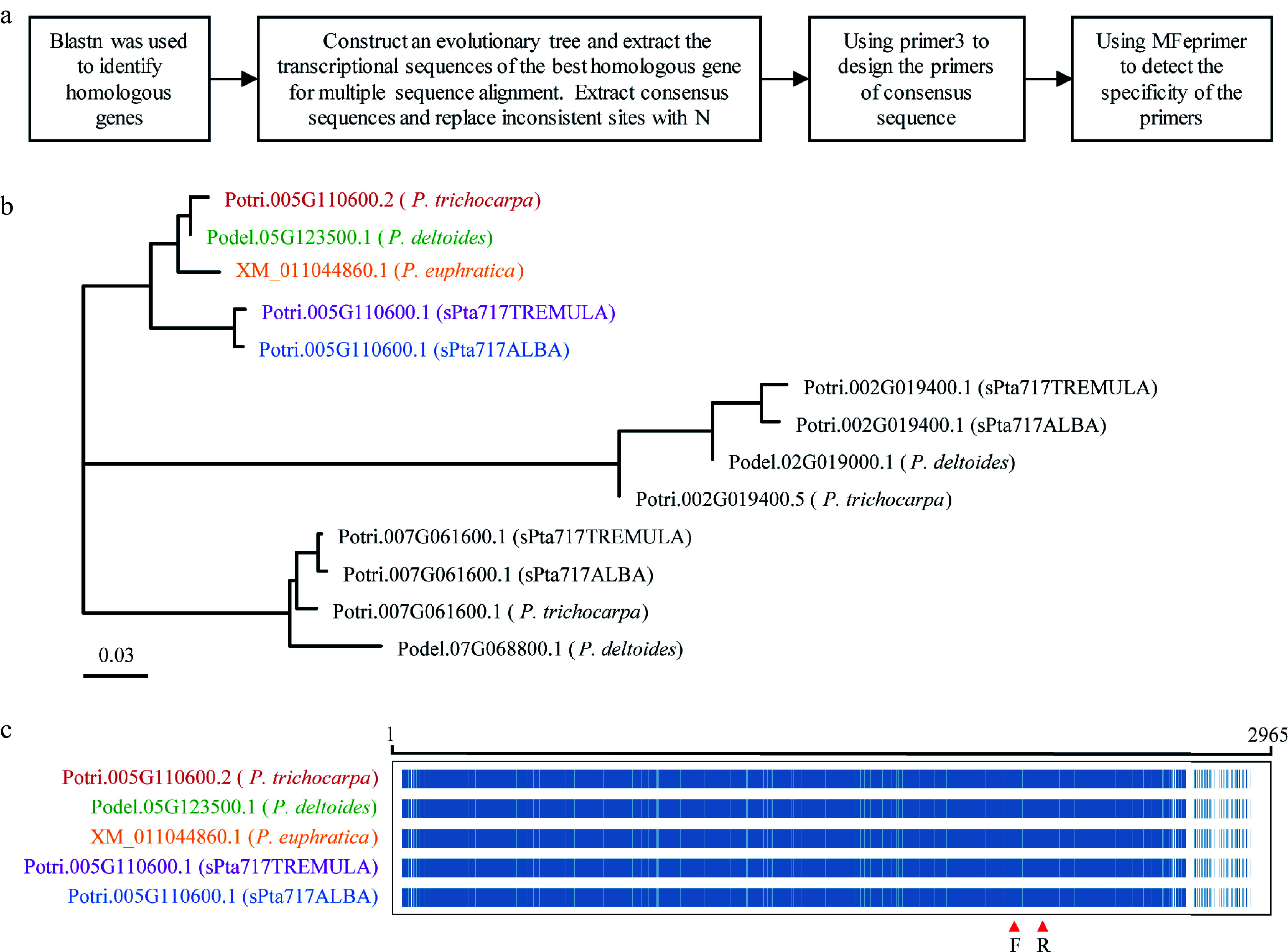
RT-qPCR primer design. This figure illustrates the process of designing RT-qPCR primers using Potri.005G110600 as a representative example. (a) Flowchart outlining the primer design process is presented. (b) Phylogenetic tree showing the target gene and homologous genes in five different *Populus* species, with color-coded gene names representing the most homologous genes to the target gene. (c) Multiple sequence alignment of transcript sequences for the target gene Potri.005G110600 and its homologous genes. The alignment highlights conserved positions in blue and non-conserved positions in white. The region indicated by red arrows corresponds to the primer binding site.

To design universal primers suitable for different poplar species, the workflow illustrated in Fig. 5a was developed. The primary transcript sequences of 18 genes in *P. trichocarpa* was used for blastn to retrieve their homologous genes from *P. trichocarpa*, *P. euphratica*, *P. deltoides*, and poplar 717. For example, when examining the primary transcript sequence of Potri.005G10600, a total of 13 homologous transcripts were retrieved from blastn searches of the five poplar genomes. A phylogenetic tree was constructed using the primary transcript sequences of Potri.005G10600 and it's 13 homologues to identify the former's orthologs (Fig. 5b). Multiple sequence alignments were performed between Potri.005G10600 and its orthologs to obtain consensus sequences for primer design (Fig. 5c). The specificity of the primers on the five poplar genomes was verified using the mfeprimer-3.2.0 software to obtain universal primers for these species. Finally, RT-qPCR primers for all 18 RGs, including 12 candidate RGs and six reported RGs, were designed according to the method described above (Supplemental Table S5).

To verify the performance of the designed primer pairs, RNA was extracted from the stems of poplar 717 tissue culture seedlings, and cDNA was synthesized through reverse transcription of RNA. The gene specificity and amplification efficiency of the designed primer pairs were then evaluated using RT-qPCR. The correlation coefficient (R^2^ values) of the 18 primer pairs in RT-qPCR amplification were all above 0.99, indicating that they had high gene specificity. The amplification efficiencies varied from 91.3% to 109.7%, all of which were in the reliable section from 90% to 115%^[58]^. These results indicated that the designed primers were suitable for further gene expression analysis.

### Validation of expression stability of candidate RGs

**Figure 6:**
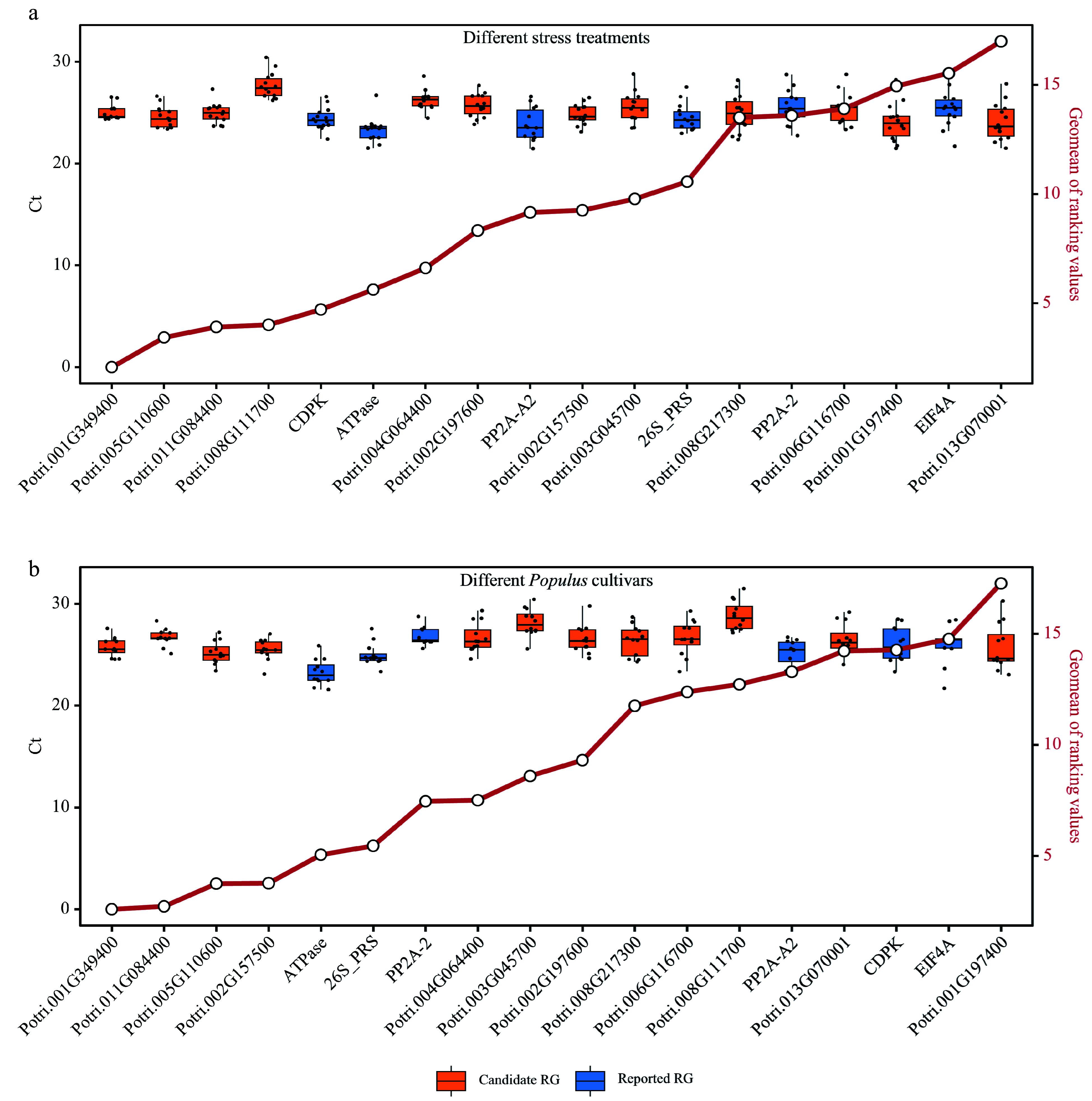
Comprehensive analysis of expression stability and Ct value distribution of reference genes (RGs). This figure presents a comprehensive evaluation of the expression stability of 18 RGs (12 candidate RGs and six reported RGs) across diverse stress treatments and *Populus* species using Ref-finder, along with the distribution of Ct values for these 18 RGs. (a) Box plots showing the Ct value distribution and integrated stability ranking of the 18 RGs under different stress treatment experiments (Supplemental Table S5). (b) Box plots depicting the Ct value distribution and integrated stability ranking of the 18 RGs in stems of five different *Populus* cultivars including *P. simonii*, *P. deltoides*, poplar 717, *P. alba*, and *P. adenopoda*. The small circles on red line indicate the integrated stability ranking, with smaller values indicating greater stability. All RT-qPCR experiments were performed with three biological replicates and two technical replicates. Each dot of the boxplot represents the average of these six replicates.

Poplar 717 plants were subjected to different stress treatments (Supplemental Table S3) to verify the expression stability of the 12 candidate RGs and six reported RGs by RT-qPCR. The expression stability of these genes was evaluated and ranked using four commonly used stability evaluation programs: delta-Ct, BestKeeper, Normfinder, and geNorm (Supplemental Dataset 5). The physiological parameters, including net photosynthetic rate (Pn), stomatal conductance (gs), intercellular CO2 (Ci), and transpiration rate (Tr) were used to monitor the stress response of poplar plants to various treatments (Supplemental Dataset 6). The comprehensive ranking was generated by computing the geometric mean (geomean) of the gene rankings. The distribution of Ct values of RT-qPCR for all RGs was concentrated closely near the mean, indicating consistent and reliable expression levels of the candidate RGs and the reported RGs (Fig. 6a). Four candidate RGs, Potri.001G349400, Potri.005G110600, Potri.011G084400 and Potri.008G111700, exhibited the highest stability and ranked at the top under all stress conditions (Fig. 6a). To further verify that the candidate RGs in this study are suitable for comparison among different poplar species, young and mature stems (IN3 and IN9) collected from five different *Populus* cultivars including *P. simonii*, *P. deltoides*, poplar 717, *P. alba*, and *P. adenopoda* grown on the campus of Huazhong Agricultural University were used. And the stability of 18 RGs in these stems was analyzed. Potri.001G349400, Potri.011G084400, Potri.005G110600 and Potri.002G157500 were among the best for stability in the stems of these cultivars (Fig. 6b). Based on the above two experiments, Potri.001G349400, Potri.005G110600, and Potri.011G084400 showed the best stability compared to other candidate RGs and reported RGs.

**Figure 7:**
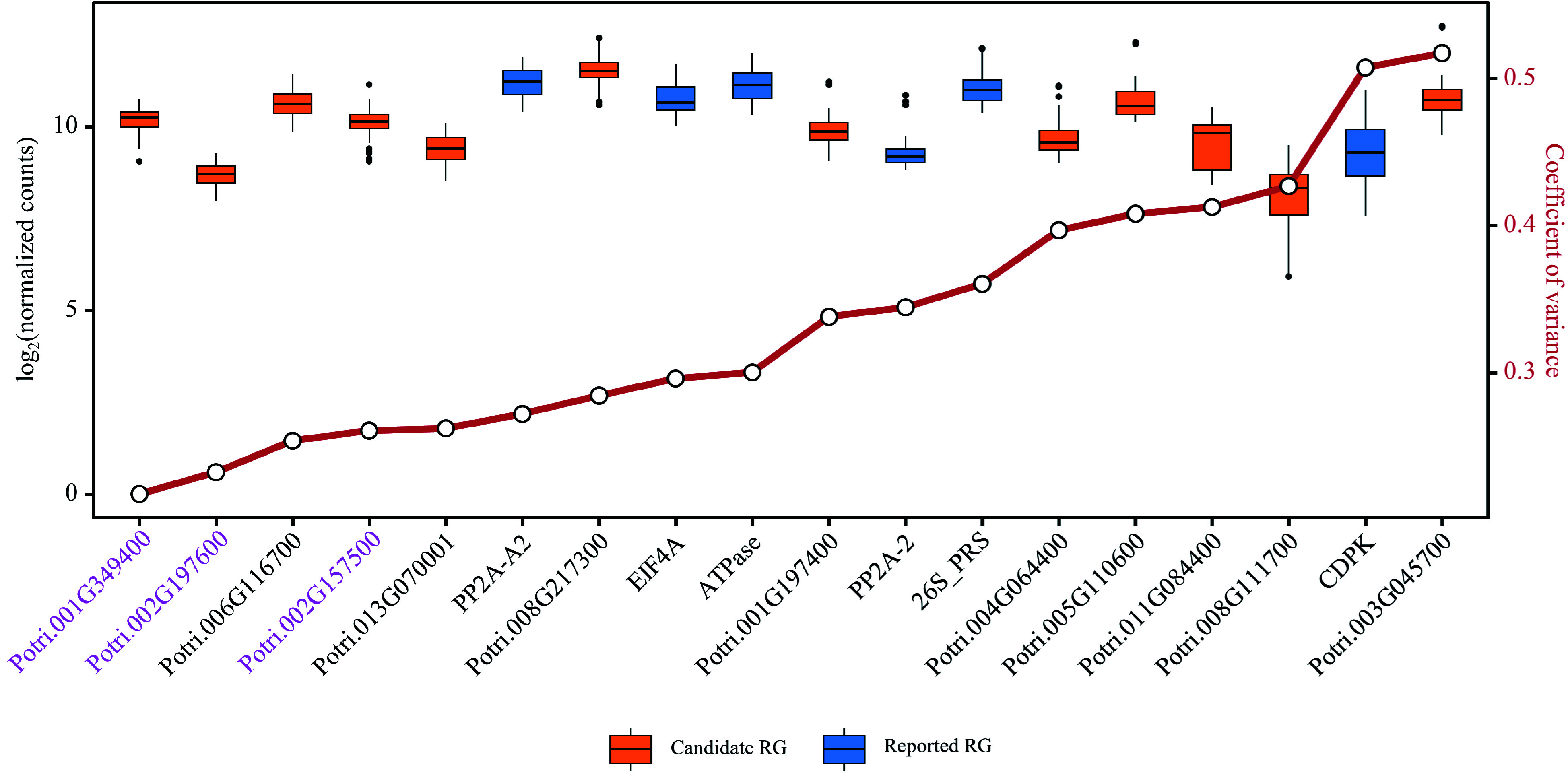
Evaluation of candidate and reported reference genes (RGs) stability based on RNA-Seq data. Box plots are employed to display the distribution of expression levels for 12 candidate RGs and six reported RGs. The small circles on the red line represents the coefficient of variance (CV) for the gene expression levels, where smaller CV values signify greater stability of gene expression. The three genes considered as novel RGs for poplar stem gene expression analysis are shown in purple.

To further verify the stability of the candidate RGs and reported RGs, a test dataset was constructed using the latest RNA-Seq data recently uploaded to the public databases, which included nine sets of poplar stem-related experiments with a total of 117 samples (Supplemental Table S4, Supplemental Dataset 7). Based on the DESeq2's median of ratios normalization method, distributions and CV values of relative expression levels of the 18 RGs in the test dataset were assessed. In total, there were six candidate RGs and three reported RGs with CV values below 0.3 (Fig. 7, Supplemental Dataset 8). Among them, Potri.001G349400, Potri.002G197600 and Potri.002G157500 can be considered as novel RGs for poplar stem gene expression analysis, and their gene annotations are respectively *CNOT2* (*CCR4-NOT transcription complex subunit 2*), *FIP37.1* (*similar to Arabidopsis FKBP12-interacting protein 37*), and *RH8* (*DEAD/DEAH box RNA helicase-like 8*). Potri.001G349400/*CNOT2* ranked first in the training dataset, RT-qPCR validation and test dataset, showing the best expression stability in stem-related samples among all poplar genes. The universal primers used to amplify Potri.001G349400/*CNOT2* were: 5'-GGTCTGACGAGCCAGCAAAGGGT-3' (forward) and 5'-CGCCGCTGCTCCTTGTGGT-3' (reverse).

## Discussion

The selection of suitable RGs for RT-qPCR normalization is crucial in gene expression analysis. While previous studies have often relied on literature-reported RGs, there is a growing recognition of the need for systematic selection of stable RGs. In this study, the aim was to identify constitutively and stably expressed genes that can serve as reliable internal controls for RT-qPCR experiments. To achieve this, both novel and reported RGs were retrieved from transcriptome datasets (Supplemental Table S1) of *Populus* at the genus level. These datasets provided a comprehensive view of gene expression under different developmental stages and abiotic stress conditions. Based on the CV values of gene expression, 12 novel candidate RGs and six reported RGs that demonstrated stable expression across these conditions were selected (Fig. 4). Furthermore, these novel RG candidate genes were evaluated using the latest poplar stem-related RNA-Seq data from public databases (Fig. 7) and three of them were suggested for poplar stem-related research, Potri.001G349400/*CNOT2*, Potri.002G197600*/FIP37.1* and Potri.002G157500/*RH8*. By systematically selecting RGs based on their expression stability, the present study provides researchers with an important resource for gene expression analysis in the stems of *Populus* and potentially other plant species. These stable RGs can serve as reliable internal controls for RT-qPCR experiments, enabling more accurate and robust gene expression studies.

Normalization methods play a crucial role in identifying internal RGs based on transcriptome data. In this study, the efficacy of several methods commonly used to identify RGs based on RNA-Seq data were evaluated. We compared four common normalization methods: FPKM, TPM, TMM, and DESeq2's median of ratios. Variation among the data is typically evaluated using metrics such as MFC (mean fold change), SD, and interquartile of expression level. CV value is a valuable metric for assessing the variability of gene expression levels relative to their mean, and it has been widely used to identify suitable RGs from transcriptome datasets^[59,60]^. In many studies, the top 1,000 expressed transcripts with the lowest CV values in contrasting environments are selected as stably expressed genes. The threshold for CV values is often set at < 16% or < 30%. Alternatively, some studies choose genes with a low CV of logarithmically transformed RPKM or transcript copy numbers, typically with a threshold of < 4%. The log_2_ (normalized values) and average CV values obtained by the five methods were evaluated. The results showed that the DESeq2's median of ratios and TMM normalization methods provided higher consistency compared to RC, FPKM, and TPM. DESeq2's median of ratios yielded the smallest CV values when tested with the reported RGs, and was therefore chosen as the normalization method for RNA-Seq data in this study. This approach ensures reliable and accurate normalization, resulting in more robust gene expression analysis.

Comprehensive analysis of RNA-Seq data obtained from different *Populus* species under different conditions facilitated the identification of highly reliable and broadly applicable candidate RGs. Only four out of 30 previously reported RGs have been identified as stably expressed genes across diverse conditions and environments based on their CV values (Fig. 3a). In addition to CV values, the expression levels of transcripts are also important considerations when selecting RGs from transcriptomic data. It is generally preferred to choose transcripts with higher expression levels as internal controls for gene quantification due to reasons of efficiency and accuracy^[24]^. In this study, both the stably expressed genes and the reported RGs generally had high gene expression levels (Fig. 3b). KEGG enrichment showed that most of these genes were involved in various key biological processes related to gene expression (Supplemental Dataset 4), providing further insights into the functional relevance of these genes. Only four reported RGs, *PP2A-2*, *EIF4A*, *CDPK*, and *ATPase*, exhibited CV values below 30%, while all the stably expressed genes had CV values < 30% (Figs 3a & 4). This indicates that the newly selected candidate genes have great potential as RGs.

In many previous studies, the selection of suitable RGs for gene expression studies are often limited to specific species and conditions^[61−64]^. This study was aimed to evaluate the novel and reported RGs at the genus level of *Populus*. Therefore, it is crucial to design primers that not only exhibit gene specificity within a particular species but also are universal among different *Populus* species. To ensure primer versatility, we employed an integrative approach to design gene-specific primers to amplify orthologous RGs from multiple species (Fig. 5). Consensus sequences based on multiple sequence alignments of candidate RGs and their orthologs from different *Populus* species were used for primer design. Subsequently, the gene specificity of the primers in five *Populus* genomes were verified using mfeprimer-3.2.0. This approach allows for consistent and reliable gene expression analysis across multiple *Populus* species, facilitating broader applicability of universal primer pairs within the genus.

By employing a combination of bioinformatics tools and analysis methods, a set of candidate RGs with high stability and applicability were successfully identified in various experimental conditions. The use of RefFinder, which integrates popular stability evaluation algorithms such as geNorm, NormFinder, BestKeeper, and delta-Ct method, allowed us to comprehensively evaluate the performance of the 18 tested genes in RT-qPCR analysis. All these RGs exhibited high stability in both young and mature stems of various *Populus* cultivars, as well as under different stress treatments in poplar 717 (Fig. 6). To further test the applicability of the candidate RGs in this study, their expression stability was evaluated using the latest RNA-Seq data from public databases (Supplemental Table S4, Fig. 7). The functional relevance of the best-performing RG, Potri.001G349400/*CNOT2* needs to be highlighted. CNOT2 is a core member of the Carbon catabolite repression4 (Ccr4)–NOT complex, which plays a crucial role in transcriptional regulation. In addition, Potri.002G197600*/FIP37.1* and Potri.002G157500/*RH8* can also be considered as novel RGs for poplar stem gene expression analysis based on their good performance in different tests in this study. In the analysis of the training and the test datasets, only two reported RGs (*EIF4A* and *ATPase*) had CV values below 0.3 in both cases. Therefore, most reported RGs are not suitable for gene expression analysis in poplar stems. The novel RGs developed in this study have strong applicability for gene expression analysis in poplar stems, but whether they apply to other tissues requires analysis based on relevant RNA-Seq data. The species involved in RNA-Seq and RT-qPCR in this study are relatively common in current poplar research and are also widely represented in the genus *Populus*, which is very useful for increasing the applicability of these RGs. When studying gene expression in other *Populus* species not included in this study, performing some amount of RNA-Seq might help improve the applicability of these RGs.

It is not cost-effective to perform RT-qPCR to test all reported RGs for each new experiment. As gene expression data based on RNA-Seq continue to accumulate from diverse experiments and species, the approach employed in this study holds promise for integrating and mining such data in a meaningful way. Identification of stable and reliable RGs will facilitate accurate and standardized gene expression analysis across different conditions and species.

## Conclusions

In conclusion, the current study presents a novel methodology for selecting stably expressed genes from transcriptome expression data, which offers several advantages over traditional approaches. By combining expression stability analysis and RT-qPCR validation, we successfully identified a set of novel and stable RGs for *Populus* stems. This methodology is targeted, convenient, and efficient, enabling the identification of new and more reliable RGs. The analytical method we developed can be applied to other plant genera and will greatly help researchers compare gene expression patterns in different species within the same genus. Furthermore, the identified RGs for stem development in *Populus* will serve as valuable tools for studying gene expression dynamics during wood formation in plants.

## SUPPLEMENTARY DATA

Supplementary data to this article can be found online.

## Data Availability

All data generated or analyzed during this study are included in this published article and its supplementary information files.
