# Supplementary material for: A protocol for identifying universal reference genes within a genus based on RNA-Seq data: a case study of poplar stem gene expression
Source: For Res (Fayettev). 2024 Jun 1;4:e021. doi: 10.48130/forres-0024-0017 (PMC11524287; doi:10.48130/forres-0024-0017)
Supplement: Supplementary file 1 — Supplementary data to this article can be found online. [file forres-0024-0017-S1.zip › 10.48130_forres-0024-0017-Suppl-TableS1.pdf]

Table S1 Source and basic information of RNA-Seq training dataset

Database	Accession number	Experiment design	Species	Sample number[a]	Reference
ArrayExpress	E-MTAB-5540	Cold, drought, heat, and salt stress	*P. trichocarpa*	27/81	[33]
ENA	ERP012528	ACC treatments	*P. tremula* × *P. tremuloides*	18/18	[38]
ENA	ERP016242	Different cell types	*P. tremula*	137/137	[32]
GEO	GSE81077	Different cell types	*P. trichocarpa*	18/24	[30]
GenBank	PRJNA471819	Different internodes	*P. trichocarpa*	18/18	[27]
SRA	SRP033626	Blister canker	*P. tomentosa*	4/4	[36]
SRA	SRP035471	miR397a over-expression	*P. trichocarpa*	12/12	[37]
SRA	SRP115696	Gravity stimulation	*P. alba* × *P. tremula*	37/37	[34]
SRA	SRP116293	Phloem, xylem	*P. euphratica*	6/13	[31]
SRA	SRP154396	Different areas	*P. nigra*	24/24	[35]

a: The numerator is the number of stem-related samples, the denominator is the total number of samples in this experiment

ArrayExpress: EMBL-EBI ArrayExpress

ENA: European Nucleotide Archive

GEO: GEO from NCBI

SRA: NCBI sequence reads archive
