# Supplementary material for: A protocol for identifying universal reference genes within a genus based on RNA-Seq data: a case study of poplar stem gene expression
Source: For Res (Fayettev). 2024 Jun 1;4:e021. doi: 10.48130/forres-0024-0017 (PMC11524287; doi:10.48130/forres-0024-0017)
Supplement: Supplementary file 1 — Supplementary data to this article can be found online. [file forres-0024-0017-S1.zip › 10.48130_forres-0024-0017-Suppl-TableS2.pdf]

Table S2 Information of reported reference genes (RGs) in *Populus*

Gene ID	Gene name	Description	Reference
Potri.001G004600	*TUA2*	Similar to alpha-tubulin 2	[47]
Potri.001G097400	*CDPK*	Similar to calcium-dependent protein kinase 1	[24]
Potri.001G166600	*U6-1*	Small nuclear ribonucleoprotein family protein	[48]
Potri.001G246900	*TUB*	Similar to Tubulin beta-2/beta-3 chain	[47]
Potri.001G262100	*SSU_S8e*	Similar to 40S ribosomal protein S8	[24]
Potri.001G309500	*ACT*	Similar to actin - garden pea	[24,46,47,48]
Potri.001G342500	*RP*	Similar to 60S ribosomal protein L27	[46]
Potri.001G418500	*UBQ*	Polyubiquitin	[48,47]
Potri.003G126800	*TUBB*	Sf124 - Tubulin	[46]
Potri.004G177500	*ATPase*	ATP synthase subunit B	[48]
Potri.005G072300	*HIS*	Histone superfamily protein	[46,47,48]
Potri.005G093900	*EIF4A*	Eukaryotic initiation factor 4A	[48]
Potri.006G170500	*RPS18*	Ribosomal protein S18	[48]
Potri.006G279600	*UBP*	Oligouridylate binding protein 1B	[48]
Potri.008G018800	*DnaJ_A2*	Similar to DnaJ protein homolog (DNAJ-1)	[24]
Potri.008G078400	*U6-2*	Small nuclear ribonucleoprotein family protein	[48]
Potri.008G092000	*TIF5A_iso*	Similar to eukaryotic translation initiation factor 5A isoform VI	[24]
Potri.009G018600	*EF1-beta*	Similar to elongation factor 1B alpha-subunit 2	[46]
Potri.009G133000	*26S_PRS*	26S proteasome regulatory subunit N10	[24]
Potri.010G055400	*PD-E1*	Similar to glyceraldehyde 3-phosphate dehydrogenase	[47]
Potri.010G127500	*PP2A-A2*	Protein phosphatase 2A subunit A2	[48]
Potri.010G243100	*DNAJ*	DNAJ homologue 2	[48]
Potri.011G132400	*bHLH*	bHLH transcription factor	[48]
Potri.012G094100	*GAPDH*	Glyceraldehyde-3-phosphate dehydrogenase	[48]
Potri.014G115100	*UBQ*	Similar to ubiquitin/ribosomal protein 27a	[46]
Potri.015G033700	*SSU_S4e*	Similar to 40S ribosomal protein S4	[24]
Potri.015G068300	*PP2A-2*	Protein phosphatase 2A-2	[48]
Potri.017G071100	*20S_PSU*	Similar to 20S proteasome beta subunit B	[24]
Potri.018G107300	*eIF5A*	Similar to eukaryotic translation initiation factor 5A isoform V.	[46]
Potri.019G099000	*LSU_L5e*	Large subunit ribosomal protein L5e	[24]
