# Supplementary material for: A protocol for identifying universal reference genes within a genus based on RNA-Seq data: a case study of poplar stem gene expression
Source: For Res (Fayettev). 2024 Jun 1;4:e021. doi: 10.48130/forres-0024-0017 (PMC11524287; doi:10.48130/forres-0024-0017)
Supplement: Supplementary file 1 — Supplementary data to this article can be found online. [file forres-0024-0017-S1.zip › 10.48130_forres-0024-0017-Suppl-TableS3.pdf]

Table S3 List of stress treatments for poplar 717

Serial No.	Stress type	Time point
1	Salt stress	2, 4 and 6 Days
2	Drought stress	5, 10 and 15 Days
3	Shade stress	5, 10 and 15 Days
4	Gravity stress (Tension wood and opposite wood)	2 Days and 2 Weeks
