# Supplementary material for: A protocol for identifying universal reference genes within a genus based on RNA-Seq data: a case study of poplar stem gene expression
Source: For Res (Fayettev). 2024 Jun 1;4:e021. doi: 10.48130/forres-0024-0017 (PMC11524287; doi:10.48130/forres-0024-0017)
Supplement: Supplementary file 1 — Supplementary data to this article can be found online. [file forres-0024-0017-S1.zip › 10.48130_forres-0024-0017-Suppl-TableS4.pdf]

Table S4 Source and basic information of RNA-Seq test datasets

Database	Accession number	Experiment design	Species	Sample number[a]	Reference
ENA	PRJEB61635	Flexure treatment	*P. tremula* × *P. tremuloides*	20/20	[53]
GEO	GSE232245	Auxin treatment	*P. tremula* × *P. alba*	32/64	[54]
SRA	PRJNA925667	*ERF15* over-expression	*P. tomentosa*	4/4	[55]
SRA	PRJNA967342	miR408 CRISPR-Cas9 and over-expression	*P. alba* × *P. glandulosa*	10/10	[56]
SRA	SRP412459	Ethylene treatment	*P. alba* × *P. glandulosa*	18/27	NA
SRA	SRP415529	*PtrVCS2* over-expression	*P. trichocarpa*	6/6	[57]
SRA	SRP430575	*PagIDD15* RNAi	*P. alba* × *P. glandulosa*	12/12	NA
SRA	SRP433298	Tension wood	*P. alba*	9/9	NA
SRA	SRP446763	Wood development	*Populus*	6/6	NA

a: The numerator is the number of stem-related samples, the denominator is the total number of samples in this experiment

ENA: European Nucleotide Archive

GEO: GEO from NCBI

SRA: NCBI sequence reads archive

NA: Not available
