# Supplementary material for: A protocol for identifying universal reference genes within a genus based on RNA-Seq data: a case study of poplar stem gene expression
Source: For Res (Fayettev). 2024 Jun 1;4:e021. doi: 10.48130/forres-0024-0017 (PMC11524287; doi:10.48130/forres-0024-0017)
Supplement: Supplementary file 1 — Supplementary data to this article can be found online. [file forres-0024-0017-S1.zip › 10.48130_forres-0024-0017-Suppl-TableS5.pdf]

Table S5 Universal RT-qPCR primers for 12 candidate reference genes (RGs) and 6 reported RGs in *Populus*

Gene ID	Primer sequence (5'→3')	Product length (bp)	Amplification Efficiency	R^2
Potri.001G349400	F: GGTCTGACGAGCCAGCAAAGGGT<br>R: CGCCGCTGCTCCTTGTGGT	214	0.968	0.993
Potri.002G157500	F: TATCCGCCGGGGATCGGTGC<br>R: CTGCTGTTGCGGAACCCTTGATTGA	81	0.913	0.998
Potri.005G110600	F: AAGGCCACGGGGTATTCGGC<br>R: CGCAAAGCACGCTTCAGGCA	146	1.005	0.995
Potri.002G197600	F: CCTGCTGGGGCATCTCTTGAACCT<br>R: GCTCTGCTATCTCCTGCTCCCGTT	157	0.993	0.994
Potri.013G070001	F: CCATATAGGGCGGCGAGTGGCA<br>R: ACTGGCAGCATTCCGGAGACAGA	118	0.984	0.995
Potri.001G197400	F: TGGGCAACCTTAGGAGTGAAGGAGGG<br>R: AAGAGGCCAGCAGTGTGACCCA	152	0.997	0.993
Potri.006G116700	F: TCGGAGCCTGCAGACATGGTGA<br>R: TTGGAGAGCAACAGGGTTGGGCTG	79	1.035	0.997
Potri.008G111700	F: CGAAGCTCCAAACCCGAGAATCGTT<br>R: AGCGCCATCAATGACATGCGGT	246	0.938	0.999
Potri.011G084400	F: TCCCGCCGCCGCTAATTTGTCT<br>R: GGGCAGAGTTGGTTTAGCCTTGACG	97	1.072	0.989
Potri.004G064400	F: TGGTGCAAAACCTCTGATGGCTCCT<br>R: TCCGAAAGCACCTCCTCACCAAGC	186	1.031	0.996
Potri.008G217300	F: TGTGTACGCAGAAGCCCCCTCA<br>R: CCTCCGGGCAATGAAATAGCGGT	224	1.097	0.998
Potri.003G045700	F: AGTGCAAGAAGAGCTGCCCCGT<br>R: CCAACCAAGCCAAGAACTTGCCC	277	0.976	0.999
Potri.015G068300	F: ATGGGGAATTTCTCCTCGTGGGGC<br>R: TGCAGCCATATTCCCGCAGCGA	199	1.018	1.000
Potri.005G093900	F: AGGAGGTCATTACGCACAAGAGCCA<br>R: ACAACCTGAAGCTCGGGTGGGA	128	0.968	0.998
Potri.001G097400	F: ACGGCGCAAATGAGCTTGGTGC<br>R: TGAGAAGGTGGCCAAACTGAGGTCAA	140	1.022	0.998
Potri.004G177500	F: GCTGCCCGAGAAGAAGTACCTGGA<br>R: TCCCTTCAATACGACCAGCACGCTC	97	0.988	0.998
Potri.010G127500	F: CCGGCGGGAACGCATTTCAGAT<br>R: CCCCAATTCCTCCGCCAATGCAA	264	0.957	0.996
Potri.009G133000	F: TTCTCGCGCAAGCACAGCACA<br>R: CGAACTTGTTTGCCGGCCATTGT	216	0.965	0.997
